# Relation between serum sclerostin and CTRP3 levels and bone mineral density in diabetic postmenopausal women

**DOI:** 10.1186/s12905-024-03311-9

**Published:** 2024-09-05

**Authors:** Inass Hassan Ahmad, Sally Said Abd Elhamed Gbr, Basma Mohamed Mohamed Ali El Naggar, Marwa Khairy Abdelwahab, Entesar Omar Ahmad El-saghier, Doaa Sayed Mohammed, Marwa Abdelmonim Mohamed, Maha S. Mohamed, Marwa Mohamed M. Ali Abd El-Rahim, Shahinaz El Attar

**Affiliations:** 1 Endocrinology and Metabolism Department, Faculty of Medicine for Girls, Al-Azhar University, Cairo, Egypt; 2 Internal Medicine Department, Faculty of Medicine for Girls, Al-Azhar University, Cairo, Egypt; 3 Rheumatology and Rehabilitation Department, Faculty of Medicine for Girls, Al-Azhar University, Cairo, Egypt; 4 Clinical Pathology Department, Faculty of Medicine for Girls, Al-Azhar University, Cairo, Egypt; 5 Medical Biochemistry and Molecular Biology Department, Faculty of Medicine for Girls, Al- Azhar University, Cairo, Egypt

**Keywords:** Type 2 diabetes mellitus, Osteoporosis, CTRP3, Sclerostin

## Abstract

**Background:**

Osteoporosis (OP) is a common finding in diabetic patients especially high-risk populations such as postmenopausal women. Sclerostin is a glycoprotein chiefly secreted by mature osteocytes and is considered a main regulator of bone formation. The C1q/TNF-Related Protein 3 (CTRP3) was found to be significantly associated with OP in postmenopausal women. The effect of type 2 diabetes mellitus (T2DM) on sclerostin and CTRP3 levels in postmenopausal women is rarely investigated. The present study aimed to assess the impact of T2DM on sclerostin and CTRP3 levels and their relation to OP in postmenopausal women.

**Methods:**

The study included 60 postmenopausal women with T2DM and 60 age-matched postmenopausal non-diabetic women. Bone mineral density (BMD) was assessed using dual energy X-ray absorptiometry (DEXA). Serum levels of sclerostin and CTRP3 were assessed using enzyme-linked immunosorbent assay (ELISA) technique.

**Results:**

Diabetic group expressed significantly higher serum levels of sclerostin when compared with non-diabetic group (110.0 ± 29.0 versus 51.5 ± 23.2 ng; *p* < 0.001). Oppositely, CTRP3 were significantly lower in the diabetic group (3.5 ± 3.5 versus 9.9 ± 3.7 ng/ml, *p* < 0.001). Multivariate logistic regression analysis identified HbA1c levels [OR (95% CI): 0.49 (0.26–0.93), *p* = 0.028], sclerotin levels [OR (95% CI): 1.06 (1.0-1.012), *p* = 0.041] and CTRP3 levels [OR (95%) CI: 1.64 (1.0-2.68), *p* = 0.047] as significant predictors of OP in diabetic patients.

**Conclusions:**

Sclerostin and CTRP3 levels are involved in OP in postmenopausal diabetic patients.

## Background

Type 2 diabetes mellitus (T2DM) is one of the most prevalent non-communicable diseases worldwide that substantially increase the risk of cardiovascular diseases, angiopathies, and various metabolic disorders [1–4]. Osteoporosis (OP) is a common finding in diabetic patients [5], especially high-risk populations such as postmenopausal women [6].

Sclerostin is a glycoprotein chiefly secreted by mature osteocytes and is considered a main regulator of bone formation through its effect on Wnt signaling [7] making its circulating level a promising biomarker for the diagnosis and prognosis of OP [8]. However, previous studies presented controversial results regarding the relation between sclerostin levels and OP [9–11].

In diabetic patients, the adipose tissue plays a vital role by secreting bioactive molecules called adipokines [12]. The chronic low-grade inflammation mediated by adipokines was shown as a potential therapeutic target for management of diabetic complications [13]. For instance, circulating levels of adiponectin are downregulated in T2DM patients. It is considered as the most beneficial adipokine in circulation, improving insulin sensitivity, endothelial functions, and inflammation [14].

The C1q/TNF-Related Protein (CTRP) family members are paralogues of adiponectin. They share favorable effects on inflammation, insulin sensitivity, and lipid metabolism [15]. Several studies observed the association between expression of CTRP family members and diabetes, coronary artery diseases, metabolic syndrome, non-alcoholic fatty liver disease, and polycystic ovary syndrome [16–18]. CTRP3 activates adenosine monophosphate-activated protein kinase (AMPK), thus enhancing insulin signaling and sensitivity [19]. Decline of circulating CTRP3 levels was linked to insulin resistance in diabetic patients and rise of its levels was noted after treatment with glucagon-like peptide-1 (GLP-1) receptor agonists [20]. Recently, serum CTRP3 was found to be significantly associated with OP in postmenopausal women [21]. The effect of T2DM on sclerostin and CTRP3 levels in postmenopausal women is rarely investigated. The present study aimed to assess the impact of T2DM) on sclerostin and CTRP3 levels and their relation to OP in postmenopausal women.

## Methods

The present cross-sectional study was conducted at Al-Azhar University Hospitals, Cairo, Egypt. The study protocol was approved by the local ethical committee and all participants provided informed consent before enrollment. The study included 60 postmenopausal women with T2DM and 60 age-matched postmenopausal non-diabetic women. Menopause was identified through a history of menstruation cessation for at least one year prior to enrollment. The diagnosis of T2DM was based on the criteria of the American Diabetes Association (ADA) [22]. We excluded women with diabetic nephropathy, morbid obesity, familial dyslipidemia, organ failure, malignancies, thyroid disorder, history of hormonal therapy, and/or associated immunological disorders. Patients were also excluded if they received medications known to impair bone metabolism e.g. thiazolidinediones or if they were under OP treatment.

Patients included in the study were submitted to careful history taking and through clinical examination. For biochemical assessment, 10 ml of venous blood were obtained from each participant after 8-hour fasting. Each collected sample was divided into two parts; 7 ml part for routine investigations and the other part was centrifuged for 10 min at 4000 rpm and was stored at – 80 ° C. Colorimetric enzymatic approaches were used for estimation of the lipid profile and blood glucose profile using Hitachi autoanalyzer 704 (Roche Diagnostics. Switzerland).

Automated Glycohemoglobin Analyzer (Tosoh Bioscience’s HLC-723GX@, Tosoh, India) was used to estimate the HbA1c in blood. Chemiluminescent immunoassay (Immulite2000, Siemens, Germany) was utilized to assess serum insulin form blood samples. The following formula was used to estimate HOMA-IR: HOMA-IR = fasting insulin (IU/mL) × plasma glucose (mg/dL)/405 [23].

Automated ELIZA (Thermo Scientific, Finland) and computer program (Scanlt for Multiscan FC 2.5.1) were used to measure serum levels of CTRP3. The device was set for CTRP3 sensitivity of 0.38 ng/ml. Serum sclerostin levels were assessed utilizing quantitative sandwich ELISA (Biomedica, Vienna, Austria).

Bone mineral density (BMD) was assessed through dual energy X-ray absorptiometry (Lunar Prodigy; General Electric Medical Systems; USA) at the level of the femur neck and L2-L4 spines. The diagnosis of OP was based on the findings of BMD, in which a T-score of less than − 2.5 was used as a cutoff value for the presence of OP; while a T-score ranging from − 1 to -2.5 was used a definition of osteopenia.

Data analysis was conducted by SPSS software, version 22.0 (SPSS Inc., Chicago, Illinois, USA). Numerical variables were expressed as number and percent and compared using t test while categorical data were presented as number and percent and compared using Fisher’s exact test or Chi-square test as appropriate. Correlation analysis was achieved using Pearson’s correlation coefficient. Binary logistic regression was used to identify predictors of OP. *p* value less than 0.05 was considered statistically significant.

## Results

The present study included 60 diabetic postmenopausal women and 60 age matched postmenopausal non-diabetic women. Clinical findings in the studied groups are shown in tabl-1. Diabetic group expressed significantly higher serum levels of sclerostin when compared with non-diabetic group (110.0 ± 29.0 versus 51.5 ± 23.2 ng, *p* < 0.001). Oppositely, CTRP3 were significantly lower in the diabetic group (3.5 ± 3.5 versus 9.9 ± 3.7 ng/ml, *p* < 0.001). BMD analysis identified osteopenia and OP in 30.0% and 46.7% respectively in the diabetic group while in the non-diabetic group, osteopenia and OP were identified in 25.0% and 25.0% of women respectively (*p* = 0.007) (Table 1).

**Table 1:** Comparison between groups according to demographic and laboratory data

	Diabetic group *n* = 60	Non-diabetic group *n* = 60	*p*-value
**Age** years	53.4 ± 7.7	56.1 ± 1.5	0.102
**SBP** mmHg	125.3 ± 15.0	112.3 ± 4.3	< 0.001
**DBP** mmHg	81.3 ± 10.1	73.7 ± 4.9	< 0.001
**BMI** kg/m^2^	33.5 ± 4.6	29.1 ± 3.0	< 0.001
**FBS** mg/dL	207.0 ± 68.0	89.2 ± 8.0	< 0.001
**PPBS** mg/dL	261.7 ± 88.0	122.2 ± 11.7	< 0.001
**HbA1c** %	9.0 ± 2.0	5.4 ± 0.3	< 0.001
**Cholesterol** mg/dL	207.3 ± 49.2	182.6 ± 23.6	< 0.001
**Triglycerides** mg/dL	168.5 ± 62.7	119.2 ± 20.5	< 0.001
**HDL** mg/dL	38.7 ± 5.8	36.9 ± 6.4	0.004
**LDL** mg/dL	139.4 ± 44.8	122.8 ± 21.3	< 0.001
**Insulin** IU/mL	15.4 ± 5.8	6.9 ± 1.5	< 0.001
**HOMA-IR**	6.6 ± 2.1	1.5 ± 0.4	< 0.001
**Sclerostin** ng/ml	110.0 ± 29.0	51.5 ± 23.2	< 0.001
**CTRP3** ng/ ml	3.5 ± 3.5	9.9 ± 3.7	< 0.001
**DEXA scan** n (%)			
Normal	14 (23.3)	30 (50.0)	0.007
Osteopenia	18 (30.0)	15 (25.0)	
Osteoporosis	28 (46.7)	15 (25.0)	

BMI: Body mass index, CTRP3: C1q/TNF-Related Protein 3, DBP: Diastolic blood pressure, DEXA: dual energy X-ray absorptiometry, FBS: Fasting blood sugar, HDL: High-density lipoprotein, HOMA-IR: Homeostatic model assessment for insulin resistance, LDL: Low-density lipoprotein, PPBS: Postprandial blood sugar, SBP: Systolic blood pressure

In the diabetic group, correlation analysis identified significant correlation between serum CTRP3 levels and age (*r* = 0.369, *p* = 0.045), SBP (*r* = 0.369, *p* = 0.045), DBP (*r* = 0.328, *p* = 0.037) and insulin (*r* = 0.612, *p* = 0.009). Also, there were significant correlations between serum sclerostin levels and HbA1c (*r*=-0.307, *p* = 0.049) and HOMA-IR (*p*=-0.732, *p* < 0.001). In the non-diabetic group, there were significant correlations between serum CTRP3 levels and age (*r* = 0.683, *p* < 0.001), SBP (*r* = 0.557, *p* < 0.001), FBS (*r* = 0.632, *p* < 0.001), HbA1c (*r*=-0.460, *p* = 0.011), HDL (*r* = 0.721, *p* < 0.001), LDL (*r*=-0.477, *p* = 0.008), insulin (*r* = 0.466, *p* = 0.010) and HOMA-IR (*r* = 0.611, *p* < 0.001). Also, there were significant correlations between sclerotin levels and age (*r*=-0.392, *p* = 0.032), DBP (*r*=-0.411, *p* = 0.024), FBS (*r*=-0.723, *p* < 0.001), PPBS (*r*=-0.510, *p* = 0.004), cholesterol (*r* = 0.40, *p* = 0.028), HDL (*r*=-0.680, *p* < 0.001), LDL (*r* = 0.548, *p* = 0.002) and HOMA-RI (*r*=-0.399, *p* = 0.029) (Table 2).

**Table 2:** Correlation between CTRP3 and sclerostin levels and clinical data

	Diabetic group				Non-diabetic group			
	CTRP3		Sclerostin		CTRP3		Sclerostin	
	*r*	*p*-value	*r*	*p*-value	*r*	*p*-value	*r*	*p*-value
Age (years)	0.369	0.045	0.095	0.619	0.683	< 0.001	-0.392	0.032
BMI	0.260	0.166	0.004	0.983	-0.300	0.107	0.055	0.773
Disease duration	-0.302	0.105	-0.009	0.964	-	-	-	-
SBP	0.369	0.045	0.171	0.367	0.557	< 0.001	-0.220	0.243
DBP	0.328	0.037	0.132	0.487	0.207	0.273	-0.411	0.024
FBS	0.137	0.470	-0.205	0.277	0.632	< 0.001	-0.723	< 0.001
PPBS	0.013	0.947	-0.131	0.490	0.207	0.271	-0.510	0.004
HbA1c	0.010	0.957	-0.307	0.049	-0.460	0.011	0.113	0.552
Cholesterol	-0.112	0.554	-0.075	0.693	-0.248	0.186	0.400	0.028
Triglycerides	0.121	0.525	0.059	0.757	0.122	0.519	0.042	0.827
HDL	0.060	0.753	-0.106	0.577	0.721	< 0.001	-0.680	< 0.001
LDL	-0.232	0.217	0.012	0.951	-0.477	0.008	0.548	0.002
Insulin	0.612	0.009	-0.286	0.265	0.466	0.010	-0.174	0.356
HOMA-IR	0.046	0.859	-0.732	< 0.001	0.611	< 0.001	-0.399	0.029

Multivariate logistic regression analysis identified HbA1c levels [OR (95% CI): 0.49 (0.26–0.93), *p* = 0.028], sclerotin levels [OR (95% CI): 1.06 (1.0-1.012), *p* = 0.041] and CTRP3 levels [OR (95%) CI: 1.64 (1.0-2.68), *p* = 0.047] as significant predictors of OP in diabetic patients (Table 3).

**Table 3:** Predictors of osteoporosis in diabetic patients

	Univariate analysis			Multivariate analysis		
	OR	95% CI	*p* value	OR	95% CI	*p* value
Age	1.16	1.04–1.31	0.011	1.12	0.87–1.45	0.36
Disease duration	1.21	1.08–1.35	0.001	1.09	0.87–1.36	0.46
HbA1c	0.53	0.38–0.75	< 0.001	0.49	0.26–0.93	0.028
Sclerostin	1.06	1.03–1.1	< 0.001	1.06	1.0-1.012	0.041
CTRP3	2.07	1.41–3.03	< 0.001	1.64	1.0-2.68	0.047

## Discussion

While the current published literature demonstrates significant association between the serum sclerostin and CTRP3 with OP, little is known about the additional impact of T2DM on these biomarkers. In the present study, multivariate logistic regression analysis identified sclerotin levels and CTRP3 levels as significant predictors of OP in diabetic patients.

Recently, emerging evidence highlighted a significant role of serum CTRP3 in regulation of bone hemostasis; the role of CTRP3 in regulation of bone structure appears to stem from its ability to maintain normal turnover of chondrocytes and cartilaginous structure through regulation of ERK1/2 and PI3K pathways [24, 25]. Thus, authors linked downregulation of serum CTRP3 to defective bone metabolism and features of OP [26]. On the other hand, the association between CTRP3 and T2DM is well-established with reported decline in serum CTRP3 levels among cases with insulin resistance and poor glycemic control [20]. Our analysis demonstrated that serum CTRP3 was independent predictor of OP in T2DM women and correlated significantly with metabolic parameters. To our knowledge, this is the first report that addressed the impact of T2DM on serum CTRP3 among women with OP.

The impact of T2DM on CTRP3 levels can be mediated through multiple mechanisms. Previous studies found an association between dyslipidemia and reduced CTRP3 levels in diabetic patients [27, 28]. Also, experimental evidence suggests a link between increased oxidative stress and inflammation related to the diabetic state and low levels of CTRP3 [29–31]. Importantly, the study of Wagner et al. [32] found that only female diabetic patients had reduced CTRP3 levels suggesting a possible relation between circulating CTRP3 levels and the hormonal status.

Sclerostin is usually secreted by osteocytes and late osteoblasts to mediate physiological bone metabolism [33]. The changes in the levels of circulating sclerostin may reflect the changes in bone activity, making it a biomarker for the diagnosis and prognosis of OP [34]. On the other hand, previous animal models demonstrated high expression of sclerostin gene, SOST, in the setting of T2DM [35]. Thus, it is logical to assume higher degree of dysregulated levels of sclerostin in patients with combined T2DM and OP. We found that serum sclerostin is a significant predictor of OP in diabetic women. In support of our conclusions, Wang and colleagues [36] showed that the combination of T2DM and OP led to higher increase in serum sclerostin than OP alone; moreover, serum sclerostin correlated with BMD parameters, HbA1c, and serum glucose level. The increased levels of sclerostin levels in diabetic patients are related to higher oxidative stress [37], poor glycemic control [38] and some genetic polymorphisms [39].

In the present study, we acknowledge the presence of some methodological limitations. The cross-sectional nature of the present study limits the validity of the observed associations and further long-term studies are still needed to confirm the sequential role of T2DM on osteoporosis biomarkers. In addition, the lack of pre-planned samples size calculation and being a single-center experience are additional limitations of the present study.

## Conclusions

In conclusion, the present study provides novel evidence about the impact of T2DM on OP biomarkers, serum CTRP3 and sclerostin. The results indicated that women with combined T2DM and OP/osteopenia exhibited more dysregulation in both biomarkers than non-diabetic women. Thus, serum CTRP3 and sclerostin can be used as biomarkers for early detection of OP in diabetic patients. Further experiments are warranted to confirm our findings and to understand the mechanistic processes behind the additional impact of T2DM on the OP biomarkers. In addition, further investigations about the link between adipose tissue and bone hemostasis are recommended.

## Data Availability

No datasets were generated or analysed during the current study.
